# Polyurethane Recycling: Thermal Decomposition of 1,3-Diphenyl Urea to Isocyanates

**DOI:** 10.3390/polym15112522

**Published:** 2023-05-30

**Authors:** Shahab Zamani, Sterre H. E. van der Voort, Jean-Paul Lange, Sascha R. A. Kersten, M. Pilar Ruiz

**Affiliations:** 1 Sustainable Process Technology, Faculty of Science and Technology, University of Twente, Drienerlolaan 5, 7522 NB Enschede, The Netherlands; 2 Shell Global Solutions International B.V., Shell Technology Centre Amsterdam, Grasweg 31, 1031 HW Amsterdam, The Netherlands

**Keywords:** polyurethanes, recycling, urea, isocyanates

## Abstract

Substituted urea linkages are formed during the production of polyurethane foam. To chemically recycle polyurethane toward its key monomers via depolymerization (i.e., isocyanate), it is essential to break the urea linkages to form the corresponding monomers, namely, an isocyanate and an amine. This work reports the thermal cracking of a model urea compound (1,3-diphenyl urea, DPU) into phenyl isocyanate and aniline in a flow reactor at different temperatures. Experiments were performed at 350–450 °C, with a continuous feed of a solution of 1 wt.% DPU in GVL. In the temperature range studied, high conversion levels of DPU are achieved (70–90 mol%), with high selectivity towards the desired products (close to 100 mol%) and high average mole balance (∼95 mol%) in all cases.

## 1. Introduction

Polyurethanes are the sixth most-produced polymer, with an annual production of more than 24 million tons worldwide in 2021 [1,2]. These polymers cover a wide range of applications, from foams for cushion materials such as couches and mattresses or insulation, to casings of electrical appliances [3,4]. However, these polymers cannot yet be properly recycled, and consequently, substantial amounts of used polyurethanes are still landfilled or incinerated for energy recovery, thus releasing toxic pollutants such as HCN, NO_x_, CO, nitriles, and TDI (toluene diisocyanate from aromatic foams) [5,6,7]. These environmental concerns and the loss of valuable products reveal the need for a more advantageous recycling approach.

The reuse of end-of-life polyurethane is most desired from an energy and economic perspective [8] but still faces challenges regarding hygiene standards for mattresses and flexible foams. Mechanical recycling is adopted in the industry as the current recycling pathway. However, due to its thermoset properties in foam applications, mechanical recycling generally results in downcycling the materials to pillow or carpet filling [3,9]. As an alternative, chemical recycling could allow the recovery of polyurethane’s monomers, polyol and diisocyanate. Past research mainly focused on retrieving the polyol monomer whilst neglecting the recovery of the diisocyanate monomer [9,10,11,12,13].

**Scheme 1:**
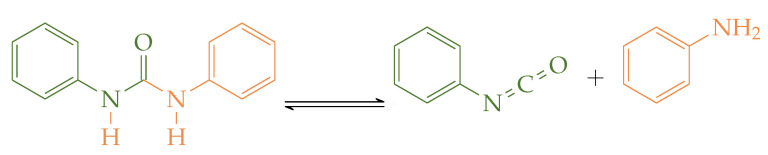
The decomposition reaction of 1,3-diphenyl urea into phenyl isocyanate and aniline.

One popular chemical recycling pathway is glycolysis/alcoholysis [3,4,10,13,14] and, more specifically, split-phase glycolysis, in which the product mixture consists of an upper apolar and a lower polar phase [13]. This apolar phase mainly consists of polyols, whereas the polar phase contains the glycol solvent, the low-molecular-weight urethanes (carbamates), urea linkages, and other aromatics [10]. By separating these two phases, the polyols are recovered with sufficient purity to be used as monomers in the polymerization of polyurethane [13]. However, the valorization of the polar phase has not yet borne fruit, as, in most cases, the usage of phosgene is needed to produce isocyanates, such as in the PUReSmart EU Horizon 2020 project [15]. The carbamate compounds present in the polar phase can be successfully cleaved to isocyanate, as reported in our previous work [16]. In this context, the present work focuses on exploring the possibility to also upgrade the urea linkages that are similarly present in the polar phase to isocyanate. To accomplish this, 1,3-Diphenyl urea (DPU) has been chosen as a model compound of such urea linkages. Its decomposition could produce phenyl isocyanate (PI) and aniline, as shown in Scheme 1.

**Scheme 2:**
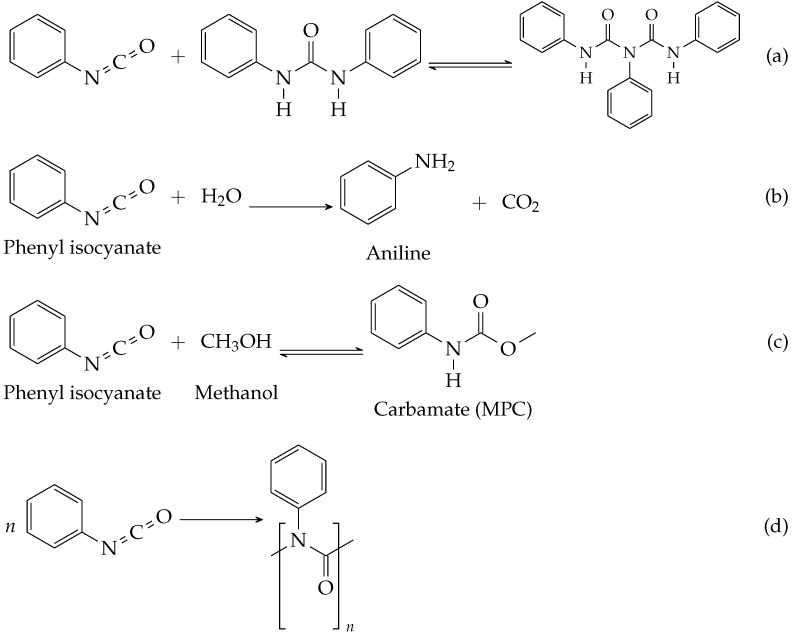

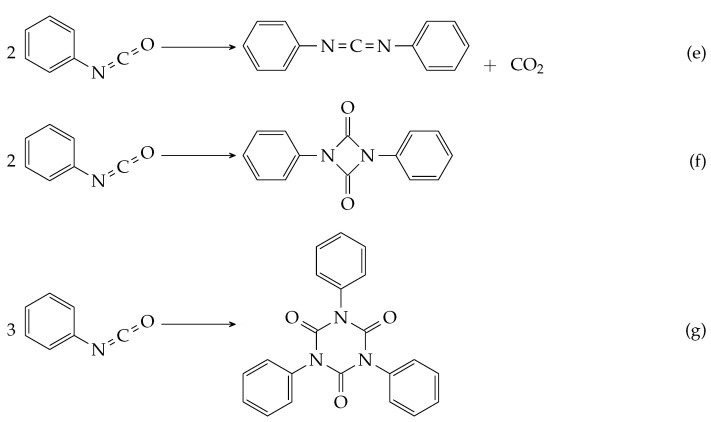
Secondary side reactions that could occur during the decomposition of 1,3-diphenyl urea. (**a**) formation of a biuret. (**b**) hydrolysis to aniline (**c**) alcoholysis to carbamate. (**d**–**g**) various oligomerizations.

However, several secondary reactions can occur with the reactive isocyanate (see Scheme 2). The phenyl isocyanate can react with unconverted DPU to form a trimeric compound with a biuret linkage (–NH-CO-NR-CO-NH–), (Scheme 2, reaction a). The addition of another phenyl isocyanate to the biuret compound can form a triuret linkage, optionally in the form of a cyclic cyanuric acid compound (not shown) [17], which can, in turn, form various higher-molecular-weight cyclic compounds [17,18]. In the presence of water, phenyl isocyanate can hydrolyze to aniline and carbon dioxide (Scheme 2, reaction b). In the presence of alcohol, a reversible reaction to a carbamate can occur (Scheme 2, reaction c) [19]. Additionally, phenyl isocyanate can react with itself in dimer, trimer, and polymer self-polymerization (Scheme 2, reaction d–g).

Whereas there are significant studies related to the decomposition of simple urea (CO(NH_2_)_2_) [18,20,21,22,23,24] and of several substituted ureas (e.g., phenylurea, dimethylurea) [19,25,26,27], studies about the decomposition of fully aromatic ureas such as DPU are scarce, limited to four studies, as shown in Table 1. Stradella and Argentero [28] reported the decomposition of DPU without solvent at >240 °C, resulting in the formation of phenyl isocyanate. However, neither conversion values nor yields were reported.

Similarly, Bennet et al. [29] performed the decomposition of DPU in solvent-free system and reported yields of phenyl isocyanate (58 mol%) and aniline (96 mol%) at 99 mol% DPU conversion at 350–370 °C in the presence of HCl. The PI yield from this study shows a significant difference with their reported conversion, indicating a loss phenyl isocyanate. This is also visible in their mole balance (not reported, but could be calculated to 78 mol%). A secondary PI reaction with itself could be considered the most logical explanation, as the authors identified a nitrogenous substance most likely to be an isocyanate polymer. Additionally, in that work, hydrogen chloride is also used next to nitrogen as a sweep gas, but the effect of this gas on the reaction was not studied.

Hoshino et al. [30] evaluated the decomposition of urea linkages in various compounds, including DPU, in several solvents at a mild temperature of 110 °C. Phenyl isocyanate was observed in mixtures with acetic acid, but was subsequently lost by hydrolysis to carbon dioxide and amine [30]. No isocyanate was observed during the DPU decomposition in various fatty acids (propionic, butyric, and caproic acid) [30]. Finally, Špírková et al. [31] reported the decomposition of DPU in 1,4-dioxane and traces of water, which presumably caused the hydrolysis of phenyl isocyanate. It can thus be noted that the decomposition of pure (solvent-free) DPU requires temperatures above at least 240 °C, as shown in Table 1. In contrast, the reaction in a solvent system can be performed at lower temperatures. However, it can also be noted that both articles that reported the reaction in a solvent do not use an inert solvent. In principle, although 1,4-dioxane can be considered inert, it had traces of water, which might cause the hydrolysis of the formed isocyanate. Therefore, it cannot be specified whether the reported hydrolysis of isocyanate is due to the decomposition reaction or the presence of traces of water. Except for the article from Bennet et al. [29], the above-mentioned literature does not report the conversion of DPU and the yields of the formed products. More generally, no study reports the mole balances of the performed experiments. Therefore, this study revisits this decomposition reaction and investigates the experimental results in terms of conversion, yields, and mole balances. The analysis method developed in our previous work [16] was used to quantify the reactants and primary products in the system. Subsequently, the effect of temperature on this reaction is studied.

## 2. Materials and Method

### 2.1. Materials

Aniline (≥99%), 1,2-dichlorobenzene (99%), dimethyl sulfoxide (>99%), 1,3-diphenyl urea (98%), diphenyl ether (99%), γ-valerolactone (98%), phenyl isocyanate (>98%), and 1-(2-pyridyl)piperazine (>99%) were purchased from Sigma-Aldrich. For the analytical equipment, acetonitrile (HPLC grade), ammonium acetate (>98%), formic acid (for LC-MS 98–100%), and water (HPLC grade) were purchased from Sigma-Aldrich.

### 2.2. DPU Solubility

The solubility of DPU at room temperature in dimethyl sulfoxide (DMSO), 1,2-dichlorobenzene, diphenyl ether, and γ-valerolactone (GVL) was determined via liquid chromatography with UV detector (LC-UV). The samples were prepared by making an over-saturated solution and subsequent filtering (Whatman 0.2 μm filter). The filtrate was diluted with a mixture of water:acetonitrile (1:1 v) to a concentration of about 15 μg/mL before analyzing the samples with LC-UV.

The solubility of DPU in DMSO was determined to be at least 189 mg/mL at room temperature; higher concentrations were not considered. In GVL, the maximum solubility of DPU was measured to be 42.6 mg/mL (4 wt.%). The solubility of DPU in dichlorobenzene and diphenyl ether (<1 mg/mL) was negligible; thus, those were not suitable as solvents for DPU.

### 2.3. Reaction Setup

**Figure 1:**
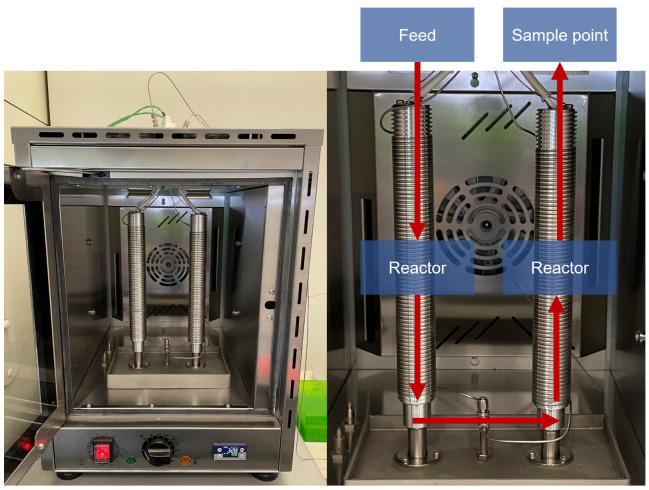
Setup for DPU decomposition and its main components.

A continuous flow unit was designed and built in-house; the diagram is shown in Figure 1. The unit consists of a pump that controls the flow throughout the system, a 1/16″ reactor tube wound in two spirals around a heating element and placed in oven, and a sample point.

The liquid is pumped from a feed flask to the first spiral, which is set to the desired temperature (350–450 °C). After a downward flow through the first spiral, a connection tube connects the first spiral to the second one, which has an upward flow toward the outlet of the second spiral. The temperature of this spiral can be set independently of the temperature of the first spiral. The flow continues in a tube after the outlet towards the sample point, where the liquid can be extracted with a needle through a septum. The cooling between the outlet of the spiral and the sampling point is sufficient for the condensation of the mixture such that the samples are extracted as a liquid, which is preferred due to safety concerns. The two spirals and the connection tube between them are insulated (not visible in Figure 1). The total volume of the setup where the reaction takes place (within the oven) is 7.25 mL. The temperature inside is measured by two thermocouples placed near the top of the spirals. The characteristics of the most essential components of the setup are shown in Table 2. Additionally, the experimental parameters used in the experiments are reported.

The experiments were run with diluted DPU to avoid precipitation. Thus, a 1 wt.% DPU in GVL solution was used as feedstock for this process. Samples were taken from the sample point (Figure 1) after a running time that corresponds to a liquid displacement of at least four reactor volumes (∼30 mL), i.e., after 30 min at a liquid flow rate of 1 mL/min.

The liquid displacement time and residence time of the compounds in the system should be selected carefully since the reactant, solvent, and products are likely to be in the gas phase at the selected operating temperatures, due to their boiling points (DPU: 262 °C; GVL: 207 °C; aniline: 184 °C; PI: 166 °C). The liquid displacement time was about 7.3 min, while the true residence time of the gaseous components is expected to be a few seconds (<2 s). After the samples were extracted via a syringe, they were prepared according to the procedure presented in the Section 2.4.

### 2.4. Analysis and Calculations

1,2-dichlorobenzene was added to the reaction mixture (1–1.5 wt.%) as an internal standard to validate the experimental procedure regarding the pipetting. This compound was used because of its chemical stability and low melting point (−17 °C).

After sample extraction, the mixture was stabilized via derivatization with a mixture of stoichiometric excess of 1-(2-pyridyl)piperazine (PP) in DMSO, as described in [16]. The samples were filtered (Whatman 0.2 µm filter) and diluted with water:acetonitrile (1:1 v) for analysis with liquid chromatography (LC) with a UV detector. Typical LC-UV spectrum obtained are shown in the Appendix A, Figure A2–Figure A4.

**Figure A1:**
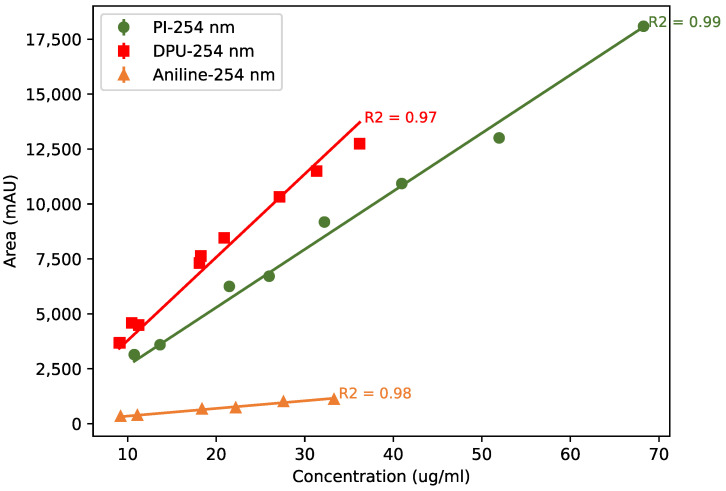
LC-UV calibration lines for the UV wavelength of 254 nm.

**Figure A2:**
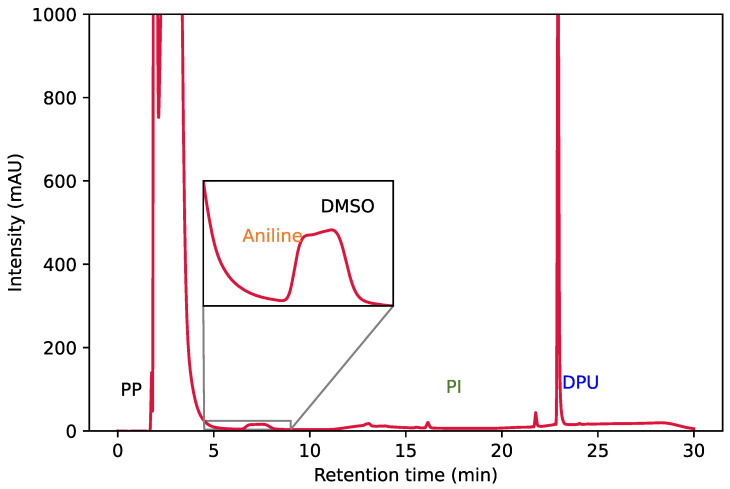
LC-UV chromatogram (254 nm) for the feedstock with 1 wt.% DPU in GVL. RT = 6.1 min (aniline), RT = 17.9 min (PI), RT = 22.8 min (DPU).

**Figure A4:**
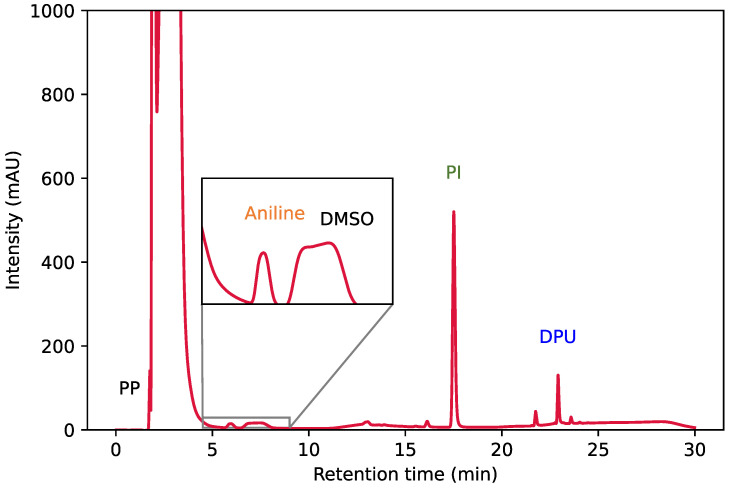
LC-UV chromatogram (254 nm) for a reaction mixture after decomposition of DPU at 450 C; 1 wt.% DPU in GVL; flowrate of 1 mL/min or liquid displacement time of 7.3 min; 1 bar. RT = 6.1 min (aniline), RT = 17.9 min (PI), RT = 22.8 min (DPU).

The LC-UV was performed with a ThermoFisher Ultimate 3000 series with an Ascentis^®^ Express RP-Amide (15 cm × 2.1 mm, 2.7 μm) HPLC column, accompanied by an Ascentis^®^ Express 90 Å RP-Amide guard column at room temperature. As a mobile phase, a gradient (0.2 mL/min) consisting of (A) 5 mM ammonium acetate with deionized water (0.1% v formic acid) and (B) acetonitrile (0.1% v formic acid) was used. The detection wavelength of the UV detector was set to measure at 254 nm. Calibration samples were prepared by diluting 1 mg/mL standard solutions of aniline, DPU, and phenyl isocyanate (PI) with a mixture of water:acetonitrile (1:1 v) (Figure A1). The reported DPU conversion $X_{A}$ was based on the molar flow rate of the reactant before and after reaction (Equation (1)). The yield for both aniline and phenyl isocyanate was calculated based on the mole of product formed and the moles fed to the systems (Equation (2)). This means that a complete conversion of DPU gives phenyl isocyanate and aniline with 100 mol% yield each. The amounts were determined based on a comparison with calibration samples of LC-UV.

(1) $$X_{A}\;\left(\%\right)\;=\;\frac{mole_{A\;fed}-mole_{A\;out}}{mole_{A\;fed}}\;\times \;100$$

(2) $$Y_{B}\;\left(\mathrm{Molar}\;\mathrm{yield};\;\mathrm{mol}\%\right)\;=\;\frac{mole_{B\;formed}}{mole_{A\;fed}}\;\times \;100$$

The selectivity to both products is reported as the yield over the conversion. Aromatic ring mole balances were calculated based on the known compounds in the system. Therefore, any missing mole balance (<100%) might indicate the presence of aromatic compounds that were not detected.

## 3. Results

The double spiral reactor was designed to mimic a plug flow reactor (PFR). The main goal of this setup was to achieve a high temperature and a short residence time to prove the selective decomposition of DPU to PI, which could not be reached in batch operation at milder temperatures for carbamate decomposition [16]. The other objective of this setup was to determine the influence of temperature on the conversion and yields during the thermal decomposition of DPU in this system.

**Figure 2:**
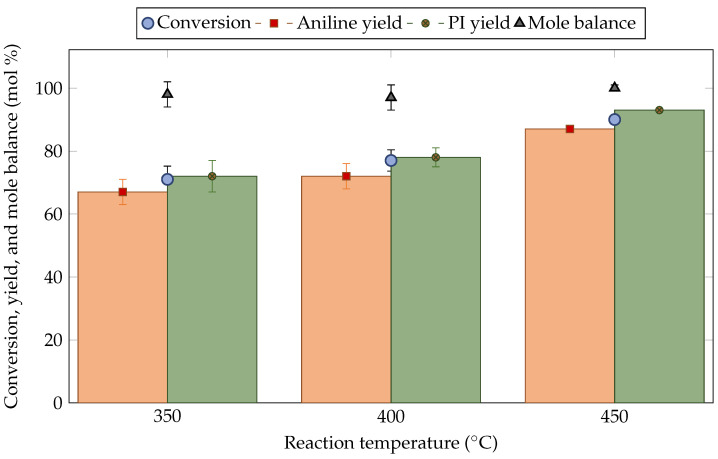
Thermal conversion of DPU to phenyl isocyanate and aniline (1 wt.% DPU in GVL; flow rate of 1 mL/min or liquid displacement time of 7.3 min; 1 bar).

The influence of temperature on the conversion, yields, and mole balance is illustrated in Figure 2. The conversion of DPU reaches values above 70 mol% under the conditions studied. It increases from 70 mol% at 350 °C to 90 mol% at 450 °C. Interestingly, at those high conversion values, the selectivity of both products (phenyl isocyanate and aniline, Scheme 1) remains close to 100 mol%.

**Figure 3:**
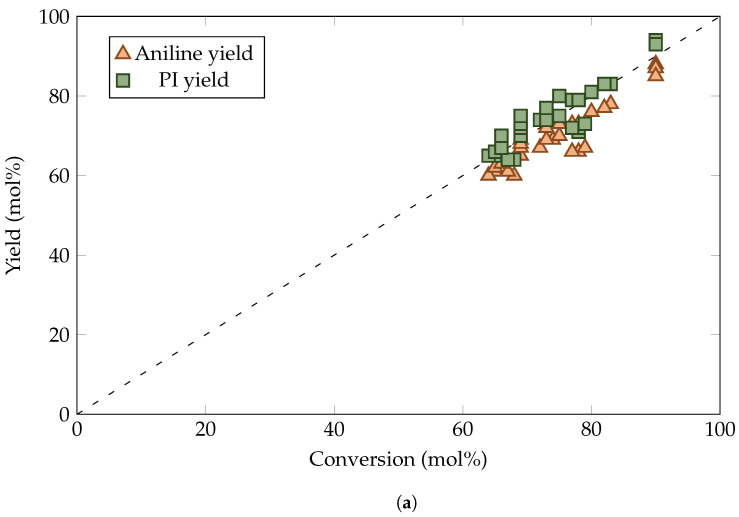

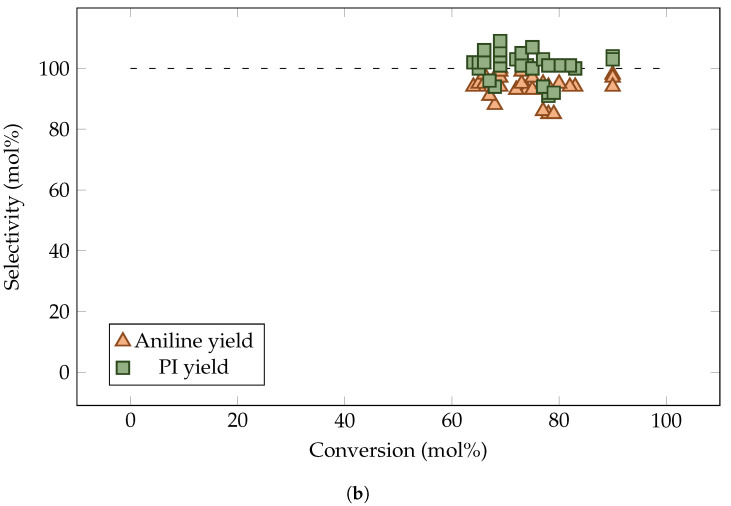
Phenyl isocyanate and aniline (**a**) yields; and (**b**) selectivities during thermal conversion of DPU (1 wt.% DPU in GVL; flowrate of 1 mL/min or liquid displacement time of 7.3 min; 1 bar.)

Figure 3a,b report the yields and selectivity of phenyl isocyanate and aniline calculated from all product samples versus the corresponding conversions. As it can be observed, the selectivity towards both products is almost 100 mol%, which indicates the absence of the secondary degradation of the phenyl isocyanate (Figure 3b). The same applies for aniline, which is formed with >90 mol% selectivity and shows no sign of consecutive degradation.

**Figure 4:**
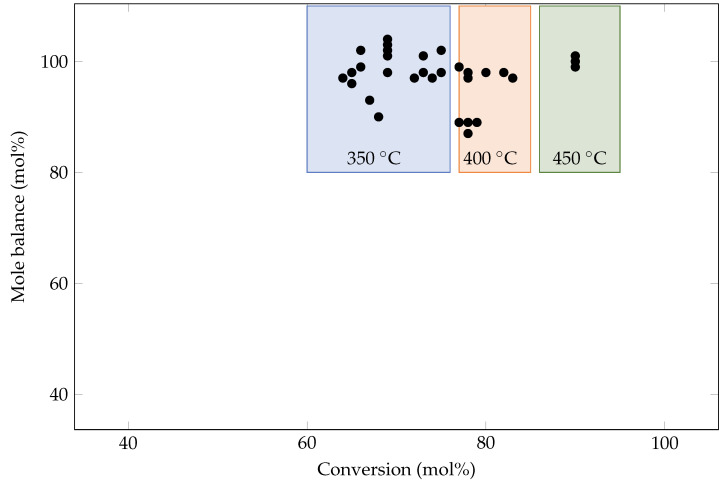
Mole balance of the quantifiable compounds and the respective conversion (1 wt.% DPU in GVL; flowrate of 1 mL/min or liquid displacement time of 7.3 min; 1 bar).

As previously mentioned, the few previous studies on this topic did not report mole balances, and in many cases, not even product yields or selectivities. Figure 4 shows the mole balance of all the experiments performed in this study. On average, it reaches 95 mol%.

In this study, we show for the first time that the conversion of phenyl urea linkages to isocyanate can be achieved at high levels of conversion, with high selectivity and closing the mole balances (all above 90 mol%), which could be a promising alternative for the valorization of one of the components derived from the glycolysis of polyurethane and thus for closing the recycling loop of those materials.

## 4. Conclusions

Cleavage of the urea linkages could be a critical step in the chemical recycling of polyurethanes towards its monomers (i.e., isocyanates). Developing a process that can achieve high yields of isocyanate, without further repolymerization, could help advance this recycling alternative. The present study has confirmed earlier claims that 1,3-diphenyl urea can decompose into aniline and phenyl isocyanate with high selectivity (close to 100 mol%) and yields (60–97 mol%) at elevated temperatures (>350 °C) when operated continuously in a diluted gas phase (1 wt.% DPU in GVL). The reaction does not suffer from consecutive degradation of the resulting isocyanate or aniline. This research opens the possibility to valorize the aromatic by-products that are obtained from PU alcoholysis or glycolysis and which contain urea components.

## Figures and Tables

**Table 1:** Overview of the literature sources about the decomposition of 1,3-diphenyl urea.

Source	Solvent	Temperature	Open/Closed	Phenyl Isocyanate
			System	Formation?
Stradella and Argentero [28]	–	>240 °C	Closed	Yes
Bennet et al. [29]	–	350–370 °C	Open	Yes (99% DPU conversion; 58% PI yield; 96% aniline yield)
Hoshino et al. [30]	Anhydrous acetic acid	110 °C	Closed	Yes
	Acetic acid + water			Yes, but it hydrolyzes
	Fatty acids			No
Špírková et al. [31]	1,4-dioxane (+ traces of water)	120 °C	Closed	Yes, but it hydrolyzes

**Table 2:** Characteristics and experimental conditions for the performed experiments of the components.

Unit	Characteristics	Experimental Conditions
Pump	Flow rate = 0.1–12 mL/min	Flow rate = 1 mL/min
Oven	T = 25–150 °C	T = 100 °C
Spirals (Each)	L = 6 m; ID = 0.8763 mm;	-
	V = 3.62 mL ^1^	
Spirals (Each)	T(max) = 500 °C	T = 350–450 °C

^1^ L = length; ID = internal diameter; V = volume.

**Figure A3:**
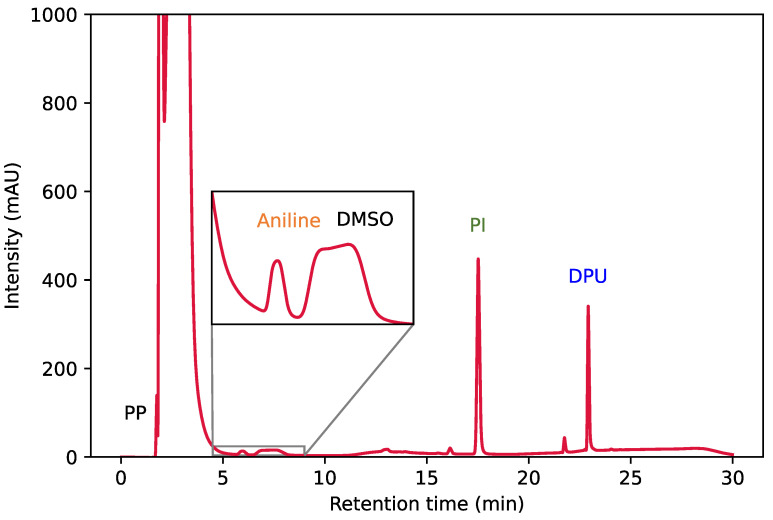
LC-UV chromatogram (254 nm) for a reaction mixture after decomposition of DPU at 350 C; 1 wt.% DPU in GVL; flowrate of 1 mL/min or liquid displacement time of 7.3 min; 1 bar. RT = 6.1 min (aniline), RT = 17.9 min (PI), RT = 22.8 min (DPU).

## Data Availability

All data obtained within this work are available at request and are to be stored within the University of Twente data archive for a minimum of 10 years.

## Abbreviations

The following abbreviations are used in this manuscript:

DMSO	Dimethyl sulfoxide
DPU	1,3-Diphenylurea
EU	European Union
GVL	γ-Valerolactone
HPLC	High-performance liquid chromatography
LC	Liquid chromatography
MDI	Methyl diphenyl diisocyanate
MPC	Methyl N-phenyl carbamate
MS	Mass spectrometer
PFR	Plug flow reactor
PI	Phenyl isocyanate
PP	1-(2-Pyridyl)piperazine
PUR	Polyurethanes
TDI	Toluene diisocyanate
UV	Ultraviolet
wt.%	Weight percent
